# Ruling out early trimester pregnancy when implementing community-based deworming programs

**DOI:** 10.1371/journal.pntd.0007901

**Published:** 2020-01-30

**Authors:** Kariane St-Denis, Brittany Blouin, Elham Rahme, Martin Casapia, Antonio Montresor, Denise Mupfasoni, Pamela Sabina Mbabazi, Theresa W. Gyorkos

**Affiliations:** 1 WHO Collaborating Centre for Research and Training in Parasite Epidemiology and Control, Department of Epidemiology, Biostatistics and Occupational Health, McGill University, Montreal, Canada; 2 Centre for Outcomes Research and Evaluation, Research Institute of the McGill University Health Centre, Montreal, Canada; 3 Asociación Civil Selva Amazoníca, Iquitos, Peru; 4 Facultad de Medicina Humana, Universidad Nacional de la Amazonía Peruana, Iquitos, Peru; 5 Department of Neglected Tropical Diseases, World Health Organization, Geneva, Switzerland; Liverpool School of Tropical Medicine, UNITED KINGDOM

## Abstract

**Background:**

Large-scale deworming programs have, to date, mostly targeted preschool- and school-age children. As community-based deworming programs become more common, deworming will be offered to women of reproductive age. The World Health Organization recommends preventive chemotherapy be administered to pregnant women only after the first trimester. It is therefore important for deworming programs to be able to identify women in early pregnancy. Our objective was to validate a short questionnaire which could be used by deworming program managers to identify and screen out women in early pregnancy.

**Methodology/Principal findings:**

In May and June 2018, interviewers administered a questionnaire, followed by a pregnancy test, to 1,203 adult women living in the Peruvian Amazon. Regression analyses were performed to identify questions with high predictive properties (using the pregnancy test as the gold standard). Test parameters were computed at different decision tree nodes (where nodes represented questions). With 106 women confirmed to be pregnant, the positive predictive value of asking the single question *‘Are you pregnant*?’ was 100%, at a ‘cost’ of a false negative rate of 1.9% (i.e. 21 women were incorrectly identified as not pregnant when they were truly pregnant). Additional questions reduced the false negative rate, but increased the false positive rate. Rates were dependent on both the combination and the order of questions.

**Conclusions/Significance:**

To identify women in early pregnancy when deworming programs are community-based, both the number and order of questions are important. The local context and cultural acceptability of different questions should inform this decision. When numbers are manageable and resources are available, pregnancy tests can be considered at different decision tree nodes to confirm pregnancy status. Trade-offs in terms of efficiency and misclassification rates will need to be considered to optimize deworming coverage in women of reproductive age.

## Author summary

To date, large-scale deworming programs have strategically targeted the high risk groups of school-age and preschool-age children in worm-endemic areas using the highly cost-effective existing infrastructure of schools. To achieve elimination of worm-attributable morbidity, however, adult populations will also need to be treated. The World Health Organization considers women of reproductive age to also be a high risk group for worm-attributable morbidity and they will be increasingly included in large-scale community-based deworming programs. Although deworming treatment is considered safe and effective at any time, it is recommended that pregnant women in the first trimester be excluded from deworming treatment. Therefore, program managers need to have a screening tool in order to rule out early trimester pregnant women during deworming program implementation. To respond to this need, we evaluated the predictive properties of a parsimonious set of questions in a study population of adult women of reproductive age in a worm-endemic region of the Peruvian Amazon. We present several question scenarios to assist program managers in using questions to rule out early trimester pregnant women. Adapted to local cultural settings, such a screening tool can optimize deworming coverage in women of reproductive age.

## Introduction

Women of reproductive age (WRA) in low- and middle-income countries are particularly vulnerable to morbidity resulting from soil-transmitted helminth infection (STH) [1–3]. It has recently been estimated that approximately 700 million WRA in over 100 countries are at risk of morbidity from these infections [4,5]. Species-specific prevalences (and intensities of infection) can vary greatly. For example, of five study populations of pregnant women recruited into randomized controlled trials in Africa and South America, the baseline prevalences of hookworm varied from 38% to 67% and for *Trichuris trichiura*, from 5% to 82% [6–10]. High STH prevalence, when combined with anemia, malnutrition and other co-occurring conditions, contribute to a high burden of disease.

Most previous research on STH infections and deworming has focused on children. Even though it is well known that worm infections cause and exacerbate anemia during the different stages of a woman’s reproductive life span, WRA have been under-studied in this context [2,11]. The resulting lack of uptake by endemic countries of deworming programs that include women of reproductive age inadvertently neglects an important cause of STH-attributable morbidity and reinforces gender inequities [3,5,12].

The deworming intervention currently used in large-scale public health intervention programs consists of a single dose of deworming medicine (either albendazole or mebendazole), repeated either once or twice a year, depending on the prevalence of STH in the area [13]. While the World Health Organization (WHO) recommends large-scale deworming programs in endemic areas for all women of reproductive age, it specifically recommends that pregnant women be treated only after the first trimester (i.e. in either the second or third trimester) [3,14]. Therefore, the challenge of large-scale community-based deworming programs is to identify pregnant women in early pregnancy in order to delay treatment. Deworming program managers would benefit from having an easily implementable and cost-efficient ‘pregnancy ruling out tool’ which would ideally rule out all women in the early stages of pregnancy while keeping in all other women. Therefore, given the emphasis on ruling out potentially pregnant women, such a tool would have the lowest false negative rate possible, even though this might increase the false positive rate.

### Determining pregnancy status

In resource-limited settings, determining pregnancy status by ultrasound, blood testing, or a urine-based pregnancy test is impractical, unfeasible and prohibitively costly, so the common practice has been to use the start date of the last menstrual period (LMP) [15]. Gestational age, based on either certain or uncertain dates of LMP, is known to be unreliable, resulting in both over- and under- estimation [16–20].

Only a small number of studies have examined the performance of a multi-item questionnaire in determining pregnancy status. In a clinic-based study sample of 283 women who had missed their periods and who had requested a pregnancy test, the authors concluded that neither a woman’s self-assessment of pregnancy nor the presence of pregnancy symptoms had a high degree of accuracy in predicting pregnancy status [21]. In a study of women seen in an emergency department setting, no combination of variables was found to be able to exclude pregnancy due to high false negative rates [22]. Several more recent publications conducted in emergency department, pre-operative and family planning settings [23–29] present conflicting results on the degree of reliability of administered questionnaires. All of these studies were conducted in clinical settings and are therefore unlikely to be generalizable to the context of community-based deworming programs.

To our knowledge, only one previous study has examined the performance of such a questionnaire in the context of women eligible for a mass treatment program (ivermectin in onchocerciasis control) in a limited resource setting [30]. Unfortunately, the published methods and results from this study in Cameroon are not easily interpretable nor replicable (e.g. results from two separate surveys were combined; not all diagnostic tests were performed on the same number of women, some having very small sample sizes; the comparator urine-based pregnancy test had a much higher threshold of human chorionic gonadotropin (hCG) than tests used today (i.e. 75 IU (international units)) with results available after two hours) and are thus unlikely to be generalizable.

The objective of the present study, therefore, was to determine the most parsimonious set of questions to identify women in early pregnancy.

## Methods

### Ethics statement

This study was approved by the Oficina del Comité Institucional de Ētica en Investigación of the Hospital Regional de Loreto "Felipe Arriola Iglesias" in Iqutios, Peru (ID 019-CIEI-2018) and by the Research Ethics Board of the McGill University Health Centre (CT1 panel; 23-04-2018), in Montreal, Canada. All participants were adults and provided written informed consent.

### Study design and study population

Data collection was completed in the 3-week period from May 29 to June 15, 2018, in Belén, an impoverished district of the capital city of Iquitos (in the department of Loreto), situated in the floodplain of the Itaya and Amazon rivers. This district has a high prevalence of STH infections in all three high risk groups (i.e. school-age children, preschool-age children and women of reproductive age, including pregnant women) [8,31,32]. A catchment area of five sub-districts was randomly selected from a list of sub-districts surrounding the three main health centers in Belén (the Centro de Salud de Belén, the Centro de Salud 9 de Octubre, and the Centro de Salud 6 de Octubre). Each sub-district had recently been mapped, such that the number of households in each neighborhood (or *manzana*) was known. The *manzana* numbers were manually entered into a database and selected by computer-generated random sequence. Every household in a selected m*anzana* was visited and every woman of reproductive age from each occupied household was invited to participate. The sample size was estimated as follows: the most recent (2016) crude birth rate in Loreto, Peru was obtained (20.83 births per 1000 persons) (World Data Atlas (Knoema) 2018). The most recent (2015) population figures for the region of Loreto were obtained to estimate the number of WRA (Citypopulation.info 2018). Using these figures, it was estimated that there would be approximately 42 pregnancies per 1000 WRA in the study area in one year. The manufacturer’s estimate of the sensitivity and specificity of the rapid pregnancy test is 100% (Core Technology Co., Ltd. 2018). Specifying an alpha of 1% and the total width of the confidence interval to be 0.03, with an expected pregnancy proportion of 0.042, the total sample size is estimated to be 1186.

Each study visit consisted of the sequential administration of the study questionnaire, followed immediately by a pregnancy test. The cross-sectional study design was chosen over that of a cohort study (where it might be perceived that a better ‘gold standard’ could be used (i.e. confirmed clinical pregnancy), for the following reasons: i) the loss to follow-up between the baseline assessments and the confirmed clinical pregnancy would likely exceed the measurement error of the currently proposed gold standard (i.e. the rapid pregnancy test); ii) some number of new pregnancies and miscarriages occurring between the baseline assessments and follow-up confirmation would be missed, contributing to measurement error; and iii) cost and feasibility issues were of concern.

The questionnaire and pregnancy tests were administered by a group of 11 research assistants (RAs) who were midwives or midwife-trainees. The RAs were trained on the administration of informed consent, the questionnaire and the pregnancy test. They worked in groups of two or three, visiting women in their own households according to a pre-set daily assignment. While obtaining informed consent, the women were asked where in their home they would be most comfortable completing the questionnaire and pregnancy test. Women were included in the study if they were between the ages of 18 and 49 years and consented to participate.

### Questionnaire

**Fig 1:**
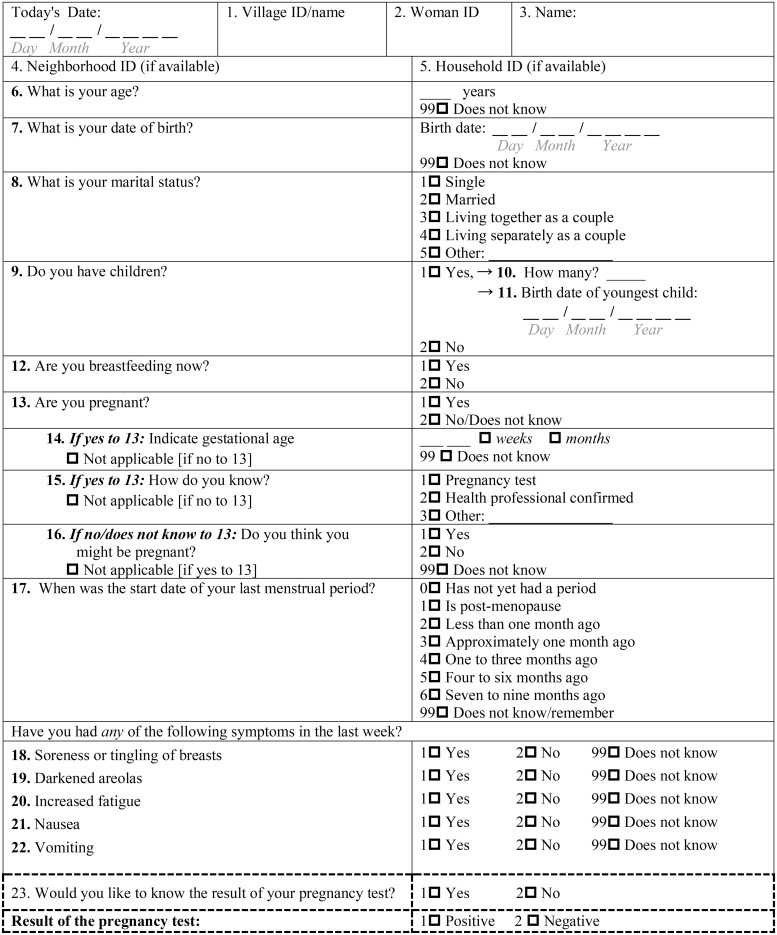
Study questionnaire (English version).

The one-page study questionnaire had five basic identifier questions (e.g. manzana number, household number), 17 questions on socio-demographic and pregnancy-related characteristics (e.g. age, birth date, marital status, number of children breastfeeding status, known or suspected pregnancy status (including gestational age and method of confirmation for women declaring they were pregnant), start date of the last menstrual cycle, and the presence/absence of breast tenderness, darkened areolas, fatigue, nausea, and vomiting) and one last question on whether or not the woman wished to receive the results of the pregnancy test (Fig 1). Several draft questions had initially been proposed by WHO and others were added by the research team based on their expertise and field experience. The questionnaire was developed in an iterative manner with several translations from English to Spanish and then back-translations from Spanish to English until the final version was confirmed. The questionnaire was designed to be administered in under 10 minutes.

### Pregnancy test

The pregnancy test (ABON hCG, Abon Biopharm (Hangzhou) Co., Ltd, China) was performed on a urine sample provided by the woman after the questionnaire was complete. The test was a rapid chromatographic immunoassay designed to detect the presence of hCG. The manufacturer reports test sensitivity to 25mIU/ML hCG with results available within 3 minutes. Elevated hCG levels can be reliably detected by this test after a minimum interval of one day following the first missed menstrual period in a woman’s cycle, with both a sensitivity and specificity of 100% (95% CI [95–100%]) and a precision level of 100% (95% CI [98%-100%]) [33]. The test was performed according to the manufacturer’s instructions and results were interpreted and recorded by the RAs on site. Women who wished to be informed of their pregnancy status were verbally provided with the results of the test.

### Data analysis

The results from both the questionnaire and the pregnancy test were entered into an electronic database by two independent research staff. Data quality was monitored daily by research personnel through field supervision and internal checks for consistency.

General socio-demographic information was summarized for the population as a whole, and stratified by pregnancy test result, to present an initial unadjusted view of the distribution of baseline variables in the study population. Comparisons were performed using independent *t*-tests for continuous variables and the chi-square test with a continuity correction for categorical variables.

Answers to the questionnaire and the pregnancy test results were summarized using univariate analyses to assess the predictive capacity of each question (answers to each question were considered as variables in the analyses) with respect to the outcome of pregnancy status. A multivariate analysis was then performed in order to assess the stability of the estimates and to identify the variables that independently had the highest predictive capacity. Odds ratios (ORs) and 95% confidence intervals (CIs) for the association between the independent predictor variables and the dependent binary variable (i.e. the pregnancy test result) were computed using logistic regression for both the univariate and multivariate analyses. Variables were removed from modeling when co-linear or sparse. Self-awareness of pregnancy status was categorized as either *knowing* one was pregnant (i.e. answering ‘Yes’ to the question: Are you pregnant?); *thinking* one was pregnant (i.e. answering ‘Yes’ to the question: Do you think you might be pregnant?); *thinking* one was *not* pregnant (i.e. answering ‘No’ to the question: Do you think you might be pregnant?); or *not knowing* whether one was pregnant (i.e. answering ‘I don’t know’ to the question: Do you think you might be pregnant?).

Diagnostic measures of sensitivity, specificity, positive predictive value (PPV) and negative predictive value (NPV) were computed for each multi-item question response against the gold standard of the binary pregnancy test result. Youden’s J statistic was then calculated to identify questions with the highest accuracy in terms of sensitivity and specificity. (The Youden’s J statistic takes into consideration both false positives (women incorrectly classified as pregnant when asked one or more questions) and false negatives (women incorrectly classified as not pregnant when asked one or more questions), and ranges from 0 (the test is completely uninformative) to 1 (the test is perfect) [34]). To further confirm potential question options and sequencing of questions, decision tree analyses were also performed. Decision trees use binary recursive splitting to partition the data into nodes, each representing a potential decision point. When used for classification, this method can predict responses associated with particular terminal node regions while minimizing classification error rates [35]. Errors in classification were described in terms of the numbers and rates of false positives and of false negatives for each decision tree node.

All statistical analyses were performed using R (version 3.4.3, R Core Team 2017, Vienna, Austria).

## Results

**Fig 2:**
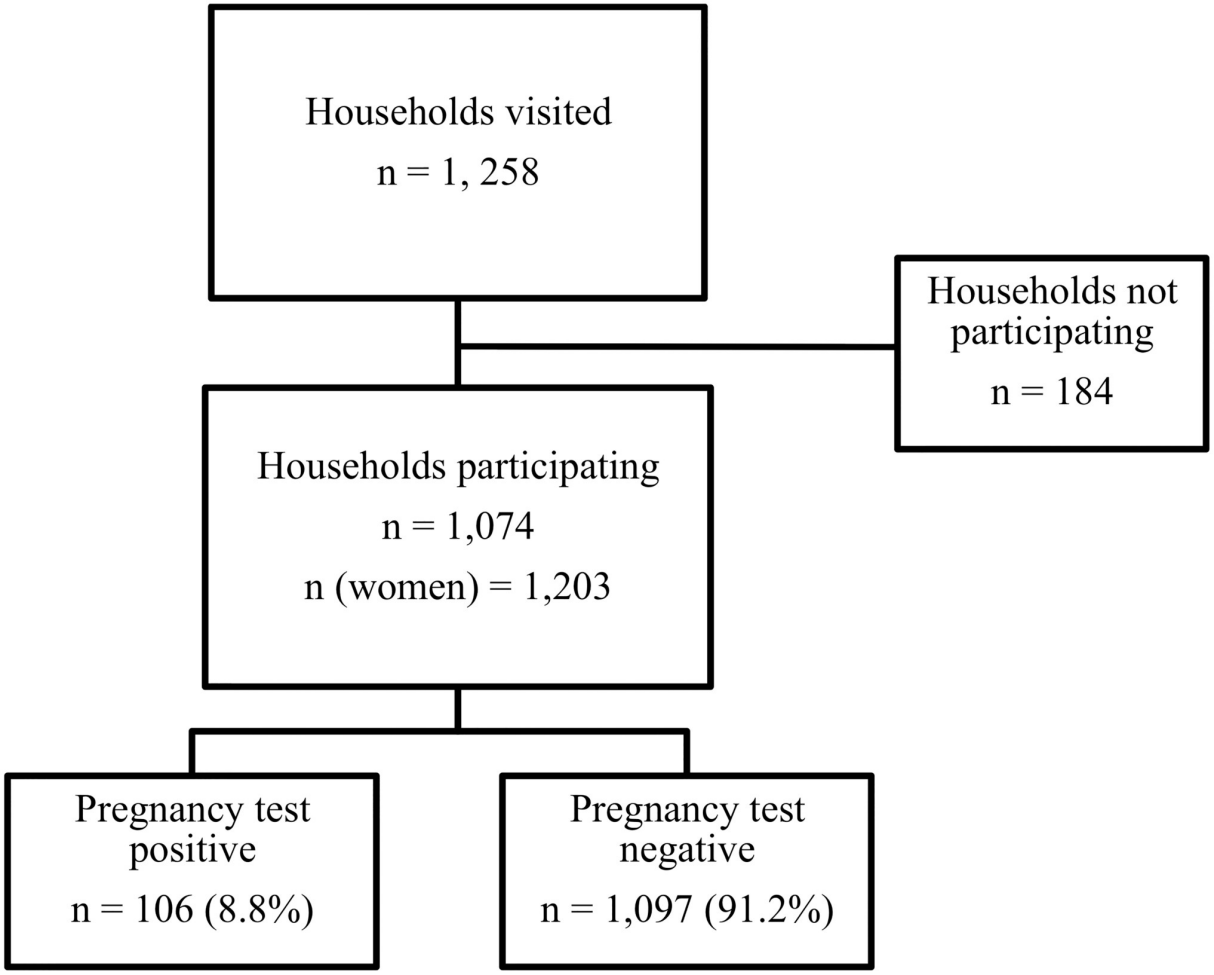
Flowchart of study population of adult women recruited from the catchment area around Iquitos, Peru, May-June 2018.

A total of 1,203 adult women consented to participate in the study. The women were recruited from 1,074 households of a total of 1,258 households (85.4%) canvassed from 182 *manzanas* located within the district of Belén. Both the study questionnaire and the pregnancy test were administered to all consenting participants. Of these, 1,097 women (91.2%) had a negative pregnancy test result and 106 women (8.8%) had a positive result (Fig 2).

Study population characteristics are summarized in Table 1. The mean age was 31.1 years (±8.7). The most commonly reported civil status was that of *conviviente*, where 63.7% of the women lived with a partner in a shared household. Other common categories included single (21.4%) and married (12.8%). Most of the women had children (86.4%), with an average of 2.4 (± 1.8) children per woman.

**Table 1:** Study population characteristics stratified by pregnancy test result (n = 1,203).

		Pregnancy test result		
	Overall	Negative	Positive	p^a^
n	**1 203**	1 097	106	
Age, mean ± SD^b^	31.12 ± 8.74	31.46 ± 8.83	27.55 ± 6.79	<0.001
Marital status, n (%)				<0.001
Single	258 (21.4)	252 (23.0)	6 (5.7)	
Married	154 (12.8)	143 (13.0)	11 (10.4)	
Partner in shared household	766 (63.7)	680 (62.0)	86 (81.1)	
Partner in separate household	24 (2.0)	21 (1.9)	3 (2.8)	
Other	1 (0.1)	1 (0.1)	0 (0)	
Has children, n (%)	1,039 (86.4)	949 (86.5)	90 (84.9)	0.756
Number of children, mean ± SD^b^	2.39 ± 1.79	2.42 ± 1.80	2.06 ± 1.65	0.048

^**a**^ p-values reported are calculated for comparisons between the group of women with a negative pregnancy test result and the group of women with a positive pregnancy test result, using the Chi-square test with continuity correction for categorical variables and independent *t*-tests for continuous variables. Statistically significant differences have p < 0.05.

^**b**^ SD: standard deviation from mean at a 95% confidence level.

The results presented in Table 1 are further stratified by the results of the pregnancy test. The marital status described as living together with a partner in a shared household was significantly more common (81.1% versus 62.0%), and that of being single significantly less common (5.7% versus 23.0%), among women who had a positive pregnancy test result. Moreover, there was a significant association between age and pregnancy status, where women with a positive test result tended to be younger than both the overall study population and women with a negative test result (p < 0.001). Women with a positive test result also tended to have had fewer children than women with a negative test result (p = 0.048).

The results of the univariate and multivariate logistic regression analyses are presented in Table 2. The following variables were found to be significant predictors of the pregnancy test results in univariate analyses: age, marital status, breastfeeding status, various levels of awareness regarding own pregnancy status, various levels of the timing of the last menstrual period, and all reported symptoms of pregnancy. In the multivariate analyses, only three questions were found to be statistically significant independent predictors of the pregnancy test results: “thinking” that she was not pregnant (adjusted odds ratio (aOR) [95% confidence interval] = 0.16 [0.05, 0.47]), date of last menstrual period < 1 month (aOR = 0.15 [0.03, 0.60]) and having darkened areolas in the past week (aOR = 9.42 [2.58, 35.34]).

**Table 2:** Association between women’s characteristics (based on an interviewer-administered questionnaire) and pregnancy test results (n = 1,203).

	Univariate		Multivariate	
Variable	OR [95% CI]	p^a^	aOR [95% CI]	p^a^
**Age**	0.94 [0.92, 0.97]	<0.001	1.00 [0.94, 1.07]	0.961
**Marital status**:				
Single	*Ref*.	*Ref*.	*Ref*.	*Ref*.
Married	3.24 [1.21, 9.59]	0.023	NA^b^	NA^b^
Partner in shared household	5.33 [2.50, 13.83]	<0.001	5.86 [1.07, 110.69]	0.099
Partner in separate household	6.02 [1.21, 24.63]	0.016	NA^b^	NA^b^
**Has children: Yes**	0.88 [0.51, 1.59]	0.646	1.45 [0.28, 10.48]	0.679
**Is currently breastfeeding: Yes**	0.21 [0.07, 0.48]	<0.001	0.64 [0.10, 2.93]	0.591
**Awareness of pregnancy status**:				
Knows she is pregnant	NA^c^	NA^c^	NA^c^	NA^c^
Thinks she is pregnant: Yes	4.64 [0.95, 17.64]	0.034	0.84 [0.08, 7.30]	0.878
Thinks she is pregnant: No	0.13 [0.05, 0.34]	<0.001	0.16 [0.05, 0.47]	0.001
Thinks she is pregnant: Does not know	*Ref*.	*Ref*.	*Ref*.	*Ref*.
**Date of last period**:				
Approximately 1 month ago	*Ref*.	*Ref*.	*Ref*.	*Ref*.
Less than 1 month ago	0.20 [0.05, 0.70]	0.013	0.15 [0.03, 0.60]	0.008
1–3 months ago	11.94 [5.04, 33.09]	<0.001	0.73 [0.15, 3.12]	0.680
4–6 months ago	37.29 [15.36, 105.92]	<0.001	1.10 [0.18, 5.51]	0.911
7–9 months ago	27.74 [10.77, 82.09]	<0.001	NA^b^	NA^b^
In menopause	NA^b^^,^^c^	NA^b^^,^^c^	NA^b^^,^^c^	NA^b^^,^^c^
Does not know/remember	1.38 [0.50, 4.13]	0.540	0.16 [0.02, 0.89]	0.051
**Experienced the following symptom in the last week**:				
Soreness of breasts	4.61 [3.03, 6.99]	<0.001	0.75 [0.18, 2.49]	0.666
Darkened areolas	65.84 [39.26, 114.44]	<0.001	9.42 [2.58, 35.34]	<0.001
Increasing fatigue	7.15 [4.64, 11.29]	<0.001	2.49 [0.84, 7.46]	0.098
Nausea	5.69 [3.55, 9.02]	<0.001	1.87 [0.39, 7.19]	0.393
Vomiting	8.66 [4.83, 15.32]	<0.001	1.38 [0.20, 7.74]	0.725

OR [95%CI], odds ratio [95% confidence interval]; aOR, adjusted odds ratio (adjusted for all other variables in the model); *Ref*, Reference category.

^a^ p-values reported are estimated using logistic regression analysis. A significance cut-off of p < 0.05 was applied. p-values inferior to 0.001 are reported as <0.001.

^b^ NA: Estimates unreliable due to having too few observations (in the category as a whole or when controlling for other variables).

^c^ NA: Estimates unreliable due to near perfect separation of the data when stratified by pregnancy test result.

* the question asking about the number of children was dichotomized (having children versus not having children) to better reflect the question which would be asked in a deworming program (rather than asking about a specific number of children)

Table 3 presents the test parameters for each question separately. While several questions had high sensitivity, specificity and predictive values, the highest Youden’s J statistic (0.802) was obtained with answering ‘Yes’ to the question *‘Are you pregnant*?’. Therefore, of the 106 truly pregnant women, this first question (Q1) would correctly identify 85 (i.e. 80.2%) women as being pregnant and they would not be treated (Table 4). Since all women identified as being pregnant were in reality pregnant, the false positive rate of this question would be 0%. However, the remaining 21 pregnant women would be in the group that answered ‘No’ to the first question (n = 1,118), leading to a false negative rate of 1.9%. In order to identify as many of these 21 pregnant women as possible so that they would be screened out from receiving the deworming treatment, further questions would need to be asked.

**Table 3:** Sensitivity, specificity, positive predictive value, negative predictive value and Youden’s J statistic, for question responses, using the pregnancy test result as the gold standard (n = 1,203).

Questions	Sensitivity (%)	Specificity (%)	PPV (%)	NPV (%)	Youden’s J
**Marital status**:					
Single	5.7	77	2.3	89.4	< 0
Married	10.4	87	7.1	90.9	< 0
Partner in shared household	81.1	38	11.2	95.4	0.191
Partner in separate household	2.8	98.1	12.5	91.3	0.009
**Has children: Yes**	84.9	13.5	8.7	90.2	< 0
**Is currently breastfeeding: Yes**	4.7	81	2.3	89.8	< 0
**Awareness of pregnancy status**:					
Knows she is pregnant	80.2	100	100	98.1	0.802
Thinks she is pregnant: Yes/does not know	66.7	82.0	6.6	99.2	0.487
Thinks she is pregnant: No	33.3	18	0.8	93.4	< 0
**Date of last period**:					
Less than 1 month ago	3.8	46.9	0.7	83.4	< 0
Not less than 1 month ago	96.2	53.1	16.6	99.3	0.493
**Experienced the following symptoms in the last week**:					
Soreness of breasts	45.3	84.8	22.3	94.1	0.301
Darkened areolas	78.3	94.8	59.3	97.8	0.731
Increasing fatigue	71.7	73.8	20.9	96.4	0.455
Nausea	32.1	92.3	28.8	93.4	0.244
Vomiting	21.7	96.9	40.4	92.8	0.186

PPV, positive predictive value; NPV, negative predictive value.

**Table 4:** Test parameters for the question: *Are you pregnant*? compared to the gold standard of the pregnancy test result (n = 1,203).

	Pregnancy test result		
*Are you pregnant*?	Positive	Negative	Totals
Yes	**85**	**0**	85
No	**21**	**1,097**	1,118
Totals	106	1,097	1,203

Test parameters: Sensitivity: 80.2%; Specificity: 100%; Positive predictive value: 100%; Negative predictive value: 98.1%; False positive rate: 0%; False negative rate: 1.9%.

The most accurate second question (Q2), based on test parameters of the 1,118 women and the re-calculated Youden’s J statistic, would be *‘Do you think you might be pregnant*?*’* (Table 5). A total of 14 of the 21 pregnant women who answered “No” to “Are you pregnant?” answered ‘Yes’ or ‘I don’t know’ to this question (n = 212). Among those answering ‘No’ to this question (n = 906), seven women would, in fact, be pregnant. Therefore, after asking these first two questions, 99 (93.4%) of the 106 truly pregnant women would have been identified as pregnant and would not be treated, while seven pregnant women (6.6%) would be among those who would have been classified as not pregnant and receive treatment (n = 906). It should be noted that, in order to identify 14 of the 21 pregnant women not identified after Q1 so that they could be excluded from treatment, 198 women would be (incorrectly) identified as being potentially pregnant and not receive treatment. As such, the addition of this question would successfully decrease the rate of false negatives (from 1.9% to 0.8%), but at the cost of increasing the rate of false positives (from 0 to 66.6%).

**Table 5:** Diagnostic parameters of individual questions among women who answered ‘No’ to the question *‘Are you pregnant*?’ (n = 1,118).

Questions	Sensitivity (%)	Specificity (%)	PPV (%)	NPV (%)	Youden’s J
**Marital status**:					
Single	4.8	77.0	0.4	97.6	< 0
Married	0	87.0	0	97.8	< 0
Partner in shared household	95.2	38.0	2.9	99.8	0.332
Partner in separate household	0	98.1	0	98.1	< 0
**Has children: Yes**	85.7	13.5	1.9	98.0	< 0
**Is currently breastfeeding: Yes**	14.3	81.0	1.4	98.0	< 0
**Awareness of pregnancy status**:					
Thinks she is pregnant: Yes/does not know	66.7	82.0	6.6	99.2	0.487
Thinks she is pregnant: No	33.3	18.0	0.77	93.4	< 0
**Date of last period**:					
Less than 1 month ago	19.0	46.9	0.7	96.7	< 0
Not less than 1 month ago	81.0	53.1	3.2	99.3	0.341
**Experienced the following symptoms in the last week**:					
Soreness of breasts	23.8	84.8	2.9	98.3	0.096
Darkened areolas	38.1	94.8	12.3	98.8	0.329
Increasing fatigue	57.1	73.8	4.0	98.9	0.309
Nausea	28.6	92.3	6.7	98.5	0.209
Vomiting	14.3	96.9	8.1	98.3	0.112

PPV, positive predictive value; NPV, negative predictive value.

To reduce the relatively large number of women who would not receive treatment (the number of false positives), while minimizing the risk of misclassifying women who were likely to be pregnant (the number of false negatives), a third question (Q3) (i.e. *‘Have you experienced darkened areolas in the past week*?’) could be asked of the 212 women who thought they might be pregnant or didn’t know if they were pregnant on Q2 (Table 6). Seventeen women answered ‘Yes" to Q3, of which eight would be correctly identified as pregnant and all 17 excluded from treatment; 195 answered ‘No’ to Q3, who would then be classified as not pregnant (when in fact, six pregnant women are in this group) and all 195 would receive treatment. Therefore, after asking three questions, 93 truly pregnant women would be correctly excluded from treatment; 1,088 truly not pregnant women would correctly receive treatment; and 13 truly pregnant women would be inadvertently treated. So, asking Q3 as an additional question would result in adding 189 non-pregnant women to the pool of women eligible for treatment (and improve coverage rates) at the ‘cost’ of misclassifying an additional six pregnant women (who would be inadvertently treated). Compared to the previous 2-question model, this 3-question model would thus translate into a slight increase in the rate of false negatives (from 0.8% to 1.2%), while achieving a large reduction in the rate of false positives (from 66.6% to 8.8%).

**Table 6:** Diagnostic parameters of individual questions among women who answered ‘No’ to the question *‘Are you pregnant*?’ and ‘Yes/don’t know’ to the question *‘Do you think you might be pregnant*?*’* (n = 212).

Questions	Sensitivity (%)	Specificity (%)	PPV (%)	NPV (%)	Youden’s J
**Marital status**:					
Single	7.1	80.3	2.5	92.4	< 0
Married	0	83.8	0	92.2	< 0
Partner in shared household	92.9	36.4	9.4	98.6	0.293
Partner in separate household	0	99.5	0	93.4	< 0
**Has children: Yes**	78.6	14.1	6.1	90.3	< 0
**Is currently breastfeeding: Yes**	0	82.3	0	92.1	< 0
**Date of last period**:					
Less than 1 month ago	14.3	50.0	2.0	89.2	< 0
Not less than 1 month ago	85.7	50.0	10.8	98.0	0.357
**Experienced the following symptoms in the last week**:					
Soreness of breasts	35.7	82.8	12.8	94.8	0.185
Darkened areolas	57.1	95.5	47.1	96.9	0.526
Increasing fatigue	57.1	69.2	11.6	95.8	0.263
Nausea	35.7	86.9	16.1	95.0	0.226
Vomiting	14.3	95.5	18.2	94.0	0.098

PPV, positive predictive value; NPV, negative predictive value.

**Fig 3:**
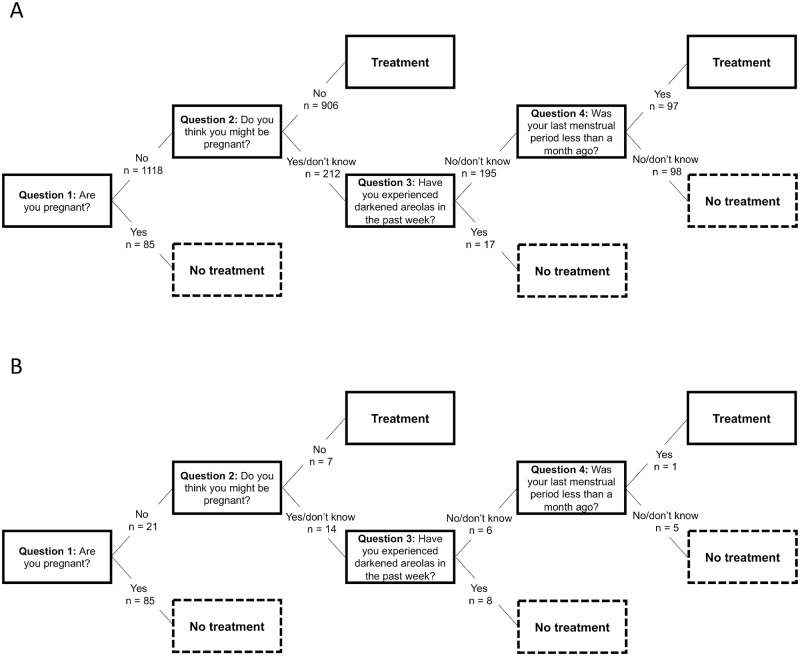
Decision trees illustrating one question sequence with high accuracy in identifying pregnant women and the corresponding deworming treatment option, where numbers indicate the decision for deworming treatment or exclusion from deworming treatment at each node, starting with the total population (n = 1,203) in panel 3A and then for pregnant women (n = 106) in panel 3B.

The misclassification in terms of the rate of false negatives can be reduced by asking a fourth question (Q4) (Table 7): either: *‘Was your last menstrual period less than one month ago*? (and if the answer is ‘No’ or ‘I don’t know’, identifying five of the six pregnant women misclassified on Q3, resulting in a false negative rate of 0.8% and a false positive rate of 51.0%) or *‘Have you experienced nausea in the past week*?*’* (and, if the answer is ‘Yes’, identifying three of the six pregnant women misclassified on Q3, resulting in a false negative rate of 0.9% and a false positive rate of 25.6%). The fourth question (and whether to use a fourth question at all) should thus be selected based on the relative importance of minimizing the rate of false negatives. The different sequences of questions, in the form of decision trees, are illustrated in Figs 3A/3B and 4A/4B.

**Table 7:** Diagnostic parameters of individual questions among women who answered ‘No’ to the question *‘Are you pregnant*?’, ‘Yes/don’t know’ to the question *‘Do you think you might be pregnant*?*’*, and ‘No/don’t know’ to the question *‘Have you experienced darkened areolas in the past week*?*’* (n = 195).

Questions	Sensitivity (%)	Specificity (%)	PPV (%)	NPV (%)	Youden’s J
**Marital status**:					
Single	16.7	80.4	2.6	96.8	< 0
Married	0	83.1	0	96.2	< 0
Partner in shared household	83.3	36.5	4.0	98.6	0.198
Partner in separate household	0	100	NA	96.9	0
**Has children: Yes**	83.3	13.8	3.0	96.3	< 0
**Is currently breastfeeding: Yes**	0	83.6	0	96.3	< 0
**Date of last period**:					
Less than 1 month ago	16.7	49.2	1.0	95.9	< 0
Not less than 1 month ago	83.3	50.8	5.1	99.0	0.341
**Experienced the following symptoms in the last week**:					
Soreness of breasts	33.3	83.1	5.9	97.5	0.164
Increasing fatigue	50.0	68.8	4.8	97.7	0.188
Nausea	50.0	87.3	11.1	98.2	0.373
Vomiting	0	95.2	0	96.8	< 0

PPV, positive predictive value; NPV, negative predictive value.

**Fig 4:**
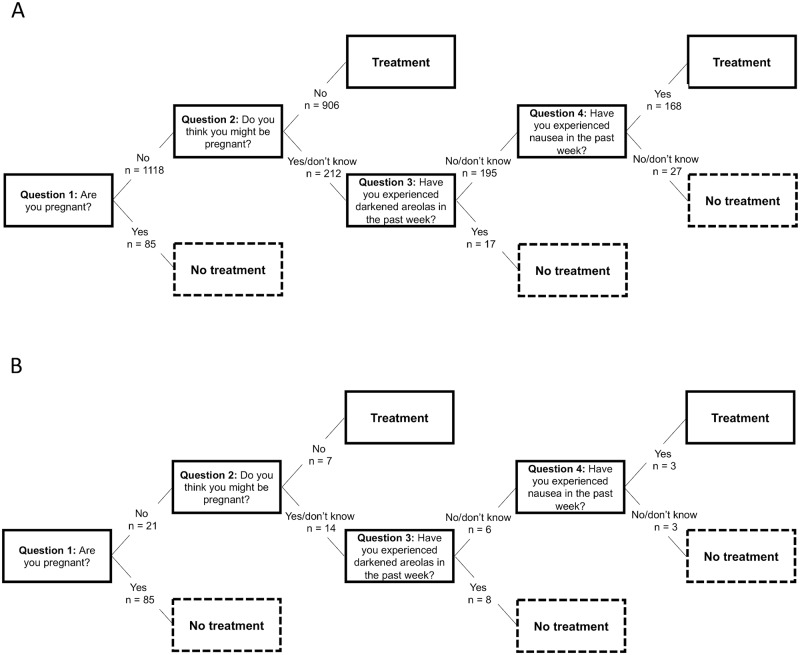
Decision trees illustrating an alternative question sequence with high accuracy in identifying pregnant women and the corresponding deworming treatment option, where numbers indicate the decision for deworming treatment or exclusion from deworming treatment at each node, starting with the total population (n = 1,203) in Panel 4A and then for pregnant women (n = 106) in panel 4B.

Additional analyses were conducted to examine whether alternative questions were more predictive of pregnancy in two distinct subgroups: 1) women who were currently breastfeeding and 2) women who had children. These did not provide any increased predictive accuracy. Subgroup analyses were also conducted to explore potential reasons for questionnaire responses (e.g. whether being younger, single or primiparous could explain a difference in responses). These analyses offered no additional insights.

## Discussion

Increasingly, girls and women of reproductive age will be included in large-scale deworming programs and it will be important to effectively rule out those who are in the early stages of pregnancy. While it is unlikely that all pregnant women will be able to be screened out unless a pregnancy test is administered, we have shown that by asking one or two questions, false negative rates can be minimized. Increases in positive predictive values (and decreases in the rate of false negatives) can only be achieved by adding one or more questions, which inevitably leads to misclassification in terms of an increase in the rate of false positives (i.e. more women would be screened out as being pregnant when they are truly not pregnant). As such, this trade-off must be balanced in light of the risks of inadvertently administering deworming treatment to pregnant women in their first trimester and the unrealized benefits stemming from eligible women not receiving the deworming treatment. To a certain extent, increases in the rate of false positives can be justified by decreases in the rate of false negatives given the relative importance of competing risks. Unfortunately, no objective standard currently exists to quantify this trade-off. One potential strategy to mitigate misclassification would be to administer pregnancy tests to those women who cannot be definitively classified as "pregnant" or "not pregnant" after one or two questions; however, pregnancy tests are not likely to be available in the context of large-scale deworming programs and the numbers of tests required might be quite high, making this approach infeasible.

Considerations other than the statistical approach used in this study should be taken into account during the implementation of a deworming program. Questions on sexual activity were not included in the present questionnaire because of acceptability issues. Cultural practices related to recognizing, and then disclosing, pregnancy status must also be considered [36]. In addition, the sex of the person distributing the deworming medicine may determine whether it is culturally appropriate to ask a particular question. Ideally, a qualitative study, with participation of women in each subgroup of women of reproductive age (i.e. adolescent girls, pregnant women, lactating women and other adult women), should be undertaken to ensure the acceptance of the proposed questions before the start of the program.

### Conclusion

The rationale for identifying women in early pregnancy in the implementation of deworming programs is the concern for adverse effects attributable to the deworming treatment in the first trimester of pregnancy. In large-scale deworming programs which include women of reproductive age, and where administering pregnancy tests to each woman would be prohibitively costly, this study has shown that inadvertent exposure to deworming treatment in early pregnancy can be considerably reduced by asking a parsimonious set of questions. The wording, number and order of questions may differ by cultural setting, in light of acceptability and feasibility concerns. These concerns can be appropriately captured in focus group discussions (ideally undertaken in all WRA subgroups) conducted prior to the launching of a WRA-targeted deworming program. This will not only enable adaptation of questions to context, including around norms linked to pregnancy disclosure, but it will also inform relevant and appropriate training for the front line health workers who will administer the questions and distribute the deworming treatment. Where possible, inadvertent exposure to deworming treatment may be further reduced, even to zero, where pregnancy tests can be administered to smaller numbers of women after answering an initial series of questions.

## Supporting information

S1 Data Final dataset containing questionnaire answers and pregnancy test results of the 1,203 women who comprised the study population (Iquitos, Peru, May-June, 2018). (XLSX)
